# Prenatal diagnosis and pregnancy outcome analysis of thickened nuchal fold in the second trimester

**DOI:** 10.1097/MD.0000000000013334

**Published:** 2018-11-16

**Authors:** Lushan Li, Fang Fu, Ru Li, Zequn Liu, Can Liao

**Affiliations:** Prenatal Diagnostic Center, Guangzhou Women and Children's Medical Center, Guangzhou Medical University, Guangdong, P.R. China.

**Keywords:** adverse outcome, CMA, karyotype, prenatal diagnosis, thickened nuchal fold

## Abstract

To summarize the results of prenatal diagnoses and pregnancy outcomes of fetuses with thickened nuchal fold (TNF) in the second trimester.

From 2009 to 2016, we studied 72 pregnant women with fetal nuchal fold (NF) measurements over 5 mm at 14 to 19 + 6 weeks or 6 mm at 20 to 28 weeks of gestation who received prenatal diagnosis. Karyotypes were first used to detect common chromosomal diseases, and then chromosome microarray analysis (CMA) was performed if karyotypes were normal. Prognoses were followed up by documentation in the hospital or over the telephone.

In total, 12 fetuses with chromosomal defects, including 5 pathogenic copy number variants (CNVs) were detected. The risk of chromosomal defects when a TNF was associated with structural malformations (SMs) (35.5%) was much greater than that of an isolated TNF (3.7%) and a TNF associated with soft markers (0%). The rate of SMs when the NF measured ≥10 mm was greater than that NF measured 5 to 7.9 mm or 8 to 9.9 mm. Totally 27 fetuses had adverse pregnancy outcome.

A TNF is not only associated with a high risk of trisomy 21 but also with other chromosomal abnormalities, including pathogenic CNVs. The rates of SMs and adverse outcomes increase when the NF thickness increases.

## 1 Introduction

The measurement of nuchal fold (NF) thickness during the second trimester is considered to be one of the most sensitive and specific isolated ultrasound marker for the identification of suspected cases of trisomy 21.^[1,2]^ An NF measurement greater than 5 mm at 14 to 17^+6^ weeks of gestation^[3,4]^ or 6 mm at 18 to 28 weeks of gestation has been associated with a markedly increased risk for Down's Syndrome.^[5,6]^ According to the practice bulletin concerning fetal aneuploidy screening published by the American Congress of Obstetricians and Gynecologists, the likely ratio (LR) for thickened nuchal fold (TNF) is 11 to 18.6.^[7]^ Although nuchal translucence (NT) is commonly used as a first-trimester screening test, pregnant women start prenatal care visits at later stages in many developing countries or districts, and chromosomal defect screening, especially by NT measurement, is not performed. Thus, second-trimester ultrasound markers for assessment of risk, especially NF, are very important.

Despite the large amount of available data regarding the relationship between a TNF and Down syndrome, few studies have examined the relationships between a TNF and other chromosomal defects and even their outcomes. Parents with fetus with a TNF are eager to learn more about those when they are undergoing clinical consultation.

The objective of this retrospective study was to summarize the results of the prenatal diagnoses and pregnancy outcomes of fetuses with a TNF in the second trimester in a tertiary hospital in Southern China.

## 2 Materials and methods

### 2.1 Study cohort

This was a retrospective single-center case series study. 72 fetuses with an NF≥5 mm at 14 to 17^+6^ weeks of gestation^[3,4]^ or 6 mm at 18 to 28 weeks of gestation who received a prenatal diagnosis in the Prenatal Diagnostic Center, Guangzhou Women and Children Medical Center during the period 2009 to 2016 were enrolled. The study included all offspring of women who underwent fetal ultrasound at our center who were diagnosed with TNF. The inclusion criteria were as follows: single pregnancy; healthy pregnant women with detailed medical history who were willing to follow up; consistent fetal gestational weeks and gestational weeks according to ultrasound prediction.

**Figure 1:**
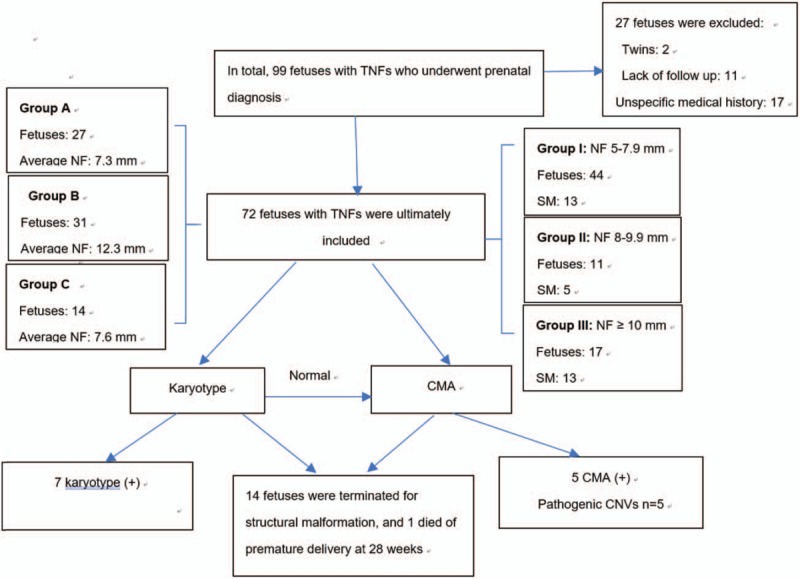
Flow chart of our study.

Patients who received amniocentesis or cordocentesis underwent a chromosome karyotype analysis, and if these cytogenetic analyses showed a normal result, a chromosome microarray analysis (CMA) was performed. Some cases associated with structural malformations (SMs) underwent a direct CMA without cytogenetic analyses (Fig. 1). The study protocol was approved by the Institutional Ethics Committee of the Guangzhou Women and Children's Hospital, China, and approved by the ethics committee of our institution.

**Figure 2:**
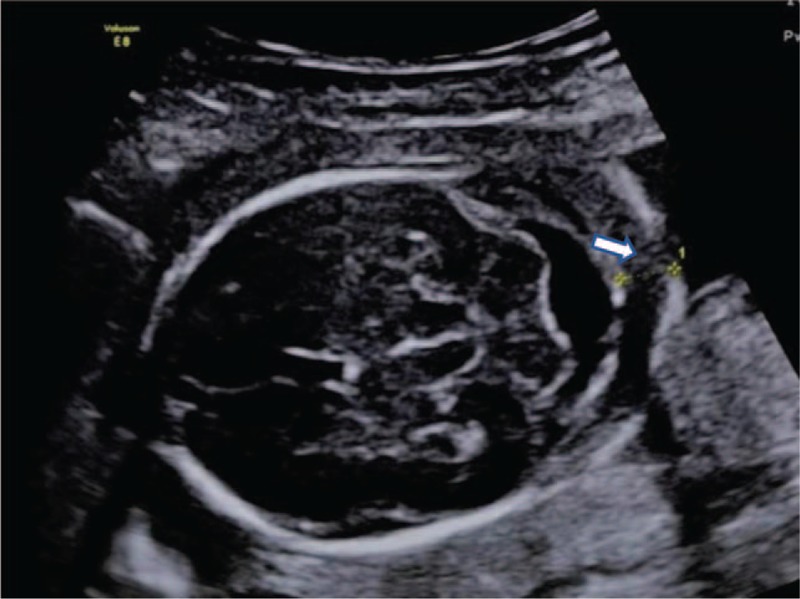
White arrow shows the NF thickness measurement in the transcerebellar plane. NF = nuchal fold.

All NF measurements were performed in the transverse plane of the fetal head, slightly removed from the biparietal diameter, which includes the cerebellum, occipital bone, and cavum septum pellucidum (Fig. 2). The NF was measured using calipers between the outer edge of the occipital bone and the outer edge of the skin.^[8,9]^ To increase the measurement accuracy, each patient underwent 2 independent measurements by 2 sonographers. To decrease intra-observer variability, the mean NF measurement obtained from these images was recorded. Fetuses with an NF measurement greater than 5 mm at 14 to 17^+6^ weeks of gestation or 6 mm at 18 to 28 weeks of gestation were included in the study, regardless of whether they were given an NT test before.

Based on whether a TNF was observed alone or with other defects, the fetuses were divided into 3 groups: Group A—isolated TNF, included 27 fetuses; Group B—TNF associated with SMs, included 31 fetuses; and Group C—TNF associated with ultrasonographic soft markers, included 14 fetuses.

Besides, Fetuses were divided into 3 groups according to the degree of NFT: Group I—NF measuring 5 to 7.9 mm, included 44 fetuses, Group II—NF measuring 8 to 9.9 mm, included 11 fetuses, and Group III—NF measuring ≥10 mm, included 17 fetuses.

### 2.2 DNA extraction and chromosomal microarray analysis

Genomic DNA was directly extracted from the uncultured amniotic fluid and cord blood samples using QIAamp DNA Blood Mini kits (Qiagen, Germany), according to the manufacturer's protocol. DNA was quantified using a NanoDrop spectrophotometer, according to the manufacturer's instructions, and the DNA quality was checked by agarose gel electrophoresis. Only cases that passed every step of the quality control process underwent microarray analysis.

A genome-wide, high-resolution SNP array, CytoScan HD (Affymetrix, Santa Clara, CA), which includes both SNPs and oligonucleotide probes, was used. Procedures for DNA digestion, ligation, polymerase chain reaction (PCR) amplification, fragmentation, labeling and hybridization of the arrays were performed according to the manufacturer's protocols. The reporting threshold for copy number variants (CNVs) was set at 100 kb, with a marker count of ≥50. The results were analyzed using Chromosome Analysis Suite software and categorized using publicly available CNV databases and by investigating the gene content and published literature. The publicly available databases included the Database of Genomic Variants (DGV), DECIPHER database, International Standards for Cytogenomic Arrays (ISCA), Online Mendelian Inheritance in Man (OMIM), and the University of California Santa Cruz Genome Browser Database (UCSC). According to the literature,^[10,11]^ the CNVs identified using CMA were classified as pathogenic CNVs, variants of unknown significance (VOUS) or benign CNVs. Alterations coinciding with known polymorphic CNVs were interpreted as benign. A CNV was considered pathogenic when its pathogenicity was confirmed by previously published literature, it contained a pathogenic phenotype-genotype-related region listed in the databases mentioned above, or evidence suggested that the dosage sensitivity resulted in a clinical phenotype. To identify possible candidate genes within the altered chromosomal regions, disease-associated and biological analyses were performed.

We summarized all types of detected chromosomal defects.

### 2.3 Clinical follow-up assessment

The prognoses of all cases were followed up by documentation in our hospital or over the telephone. Fetuses with normal fetal ultrasound findings during pregnancy, except for a TNF, and with normal physical examinations at least 1 year after birth were assumed to be normal. Adverse pregnancy outcomes included chromosomal defects, termination of pregnancy because of SMs, premature delivery, still births, and perinatal or infant death.

### 2.4 Statistical analysis

All statistical analyses were performed using SPSS 20.0. The differences among groups were analyzed using χ2 tests. A *P* value <.05 was considered significant. Descriptive statistics (median, average, maximum, and minimum values) are used for to present the results.

## 3 Results

The median age of the pregnant women in our study was 29 (range 12–41) years old, and the procedure was performed at a median of 24 (range 16–35) weeks. Of the women who experienced adverse outcomes, 12 were carrying fetuses (16.7%) with clinically significant chromosomal abnormalities, (Table 1) and all of these women (12/27, 44.4%) elected to terminate their pregnancies. Fourteen (14/27, 51.8%) women who carried fetuses with a normal karyotype and CMA but with structural abnormalities decided to terminate their pregnancies. One TNF fetus with right pleural effusion and a small stomach bubble died during premature delivery at 28 weeks. The total number of fetuses with an adverse outcome was 27 (27/72, 37.5%).

**Table 1:**
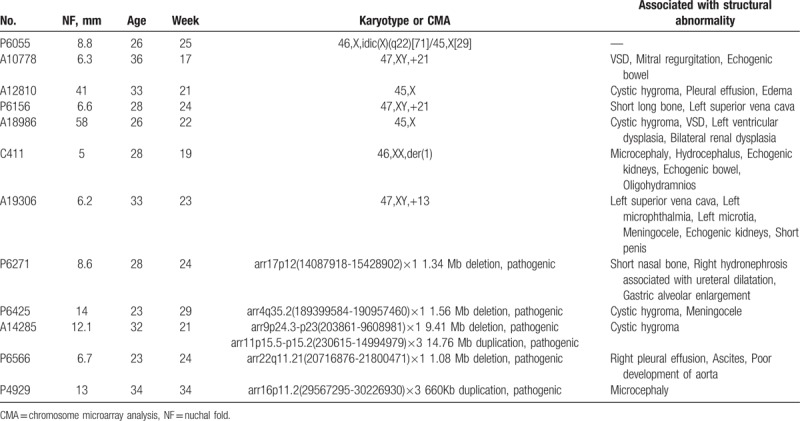
Characteristics of fetuses with chromosomal defects among the 72 fetuses with thickened nuchal fold.

**Figure 3:**
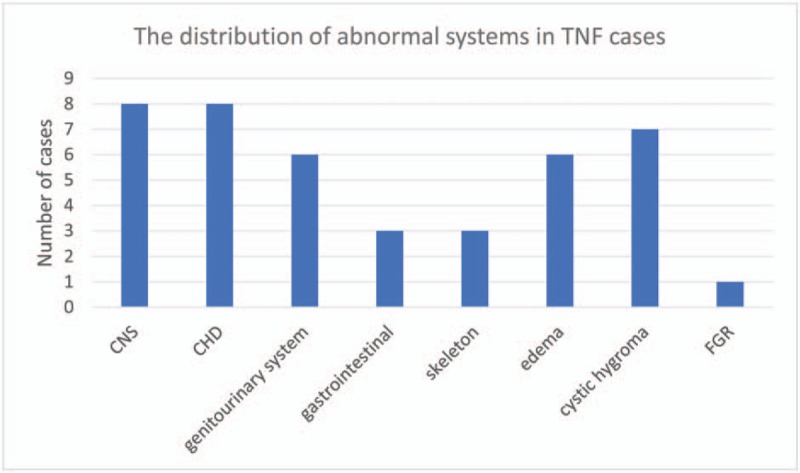
The distribution of abnormal systems in TNF cases. TNF = thickened nuchal fold.

**Figure 4:**
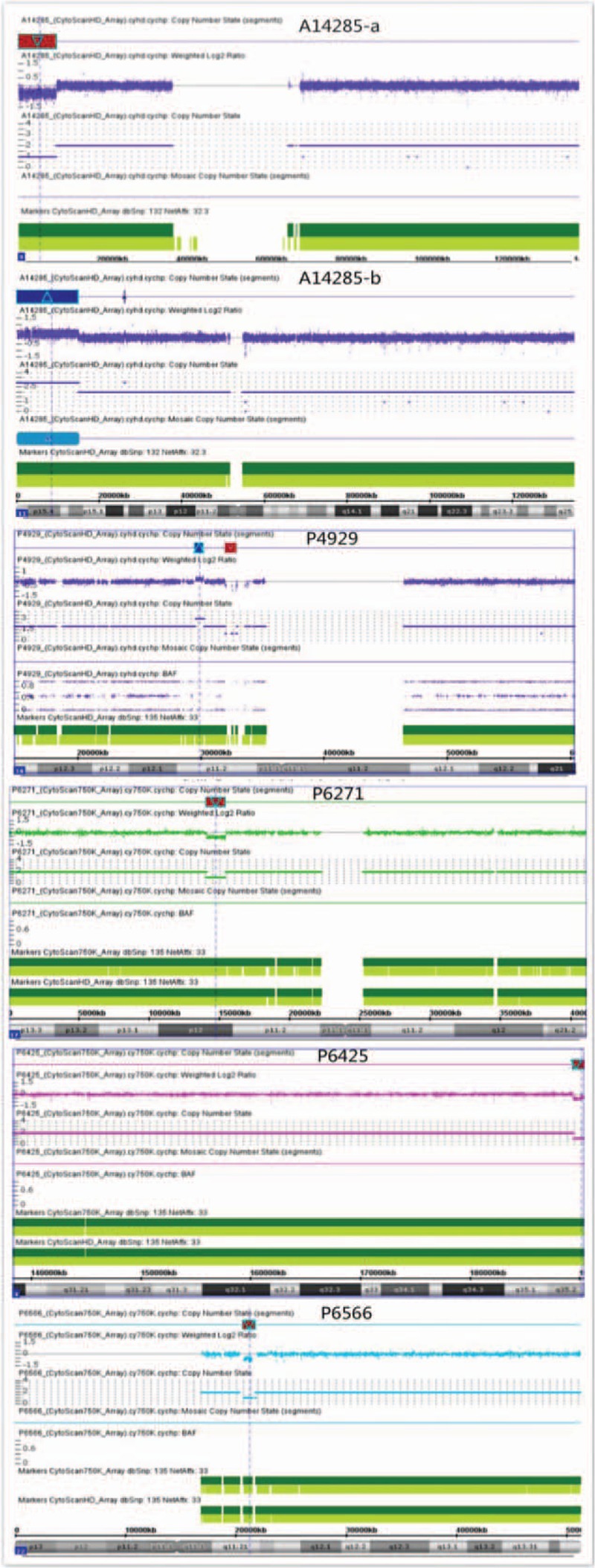
The 5 pathogenic CNVs identified in TNF fetuses. A14285-a shows a 9.41 Mb deletion in chromosome 9p24.3-p23 and A14285-b shows a 14.76 Mb duplication in 11p15.5-p15.2. P4929 shows a 660Kb duplication in chromosome 16p11.2, P6271 shows a 1.34 Mb deletion in chromosome 17p12, P6425 shows a 1.56Mb deletion in chromosome 4q35.2, P6566 shows a 1.08Mb deletion in chromosome 22q11.21. CNV = copy number variant, TNF = thickened nuchal fold.

Based on whether TNF was detected alone or in combination with other defects, fetuses were divided into 3 groups: Group A included 1 case (3.8%) of chromosome mosaicism: 46,X,idic(X) (q22)[71] /45,X[29]. Group B (in combination with other defects, Fig. 3) included 11 cases (35.5%) of chromosomal defects, including 2 cases of trisomy 21, 2 cases of Turner Syndrome, 1 case of trisomy 13, 1 case of derivative chromosome 1, and 5 cases of pathogenic CNVs (Fig. 4). Group C had no chromosomal defects. Significant differences were found between Group A and Group B and between Group B and Group C (*P* <.05) (Table 2).

**Table 2:**
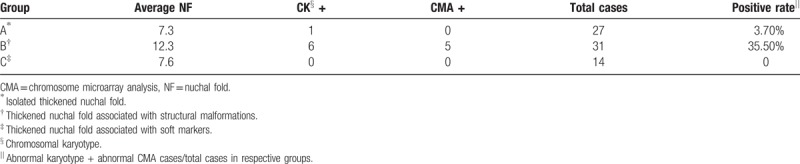
Relationship between chromosomal defects and nuchal folds (isolated or not isolated).

According to the degree of NF, fetuses were divided into 3 groups. Group I comprised 5 cases (11.4%) with chromosomal defects including 2 cases of trisomy 21, 1 case of trisomy 13, 1 case with a derivative chromosome 1 and 1 case of 22q11 microduplication syndrome. In Group I, 13 cases (29.5%) were associated with SMs, of which 11 patients chose termination, and 1 fetus died due to premature delivery. Group II comprised 2 cases (18.2%) with chromosomal defects, including 1 case with chromosome rearrangement, 46,X,idic(X) (q22)[71]/45,X[29] and 1 case with a pathogenic 17p12 microdeletion. In Group II, 5 cases (45.5%) were associated with SMs, of which 4 patients chose termination. Group III comprised 5 cases (29.4%) with chromosomal defects including 2 cases of Turner Syndrome, 1 case of a pathogenic 4q35.2 microdeletion, 1 case of both a pathogenic 9p24.3-p23 microdeletion and a pathogenic 11p15.5-p15.2 microduplication, and 1 case of 16p11.2 microduplication syndrome. In Group III, 13 cases (76.4%) were associated with SMs, of which 10 patients chose termination. The rates of SMs and adverse outcomes increased when NFT increased, especially when the NF measured greater than 10 mm (*P* <.05). No statistically significant differences were observed among the 3 groups with regard to the positive rate of chromosomal defects (*P* >.05) (Table 3).

**Table 3:**
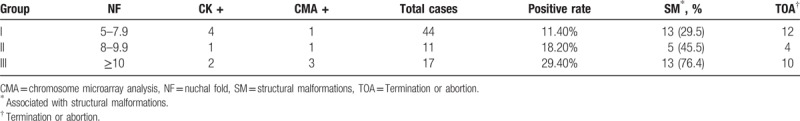
Abnormal chromosome rates among various nuchal folds.

## 4 Discussion

The physiological cause(s) of TNF is still poorly understood. In a recent study that included a large number of subjects, a correlation was found between NT and NF measurements.^[12]^ TNF may share some of the same hypotheses with increased NT. Increased NT thickness corresponds to a common phenotypic expression of genomic imbalances, such as aneuploidies (trisomy 21, 18 and 13, triploidy and X monosomy) and segmental chromosome abnormalities.^[12]^ Similar to increased NT, a TNF as an ultrasound soft marker is highly related to trisomy 21. But the relationships between TNF and pathogenetic CNVs were never reported before. In our retrospective study, 72 fetuses with TNF were investigated by karyotype and/or CMA, and the results showed trisomy 21 accounting for 2.8%, Turner syndrome accounting for 2.8%, trisomy 13 accounting for 1.4%, pathogenetic CNVs accounting for 6.9% and other chromosome aberrant accounting for 2.8%. Using NF alone with a commonly used threshold of 5 or 6 mm, reported detection rates for trisomy 21 vary widely, from 4% to 35%.^[13–16]^ The detection rate for trisomy 21 in our study was lower than the rate reported before, which is likely due to the prevalence of the NT test and Non-Invasive Prenatal Testing (NIPT) that allowed the early diagnosis and termination of trisomy 21 in the first trimester, resulting in fewer cases in the second trimester. It is obvious that regardless of aneuploidy or chromosomal structural aberrations, most of these TNF cases were associated with various structural abnormalities. Except for 1 isolated TNF case associated with chromosomal defects (46,X,idic(X) (q22)[71] /45,X[29]) and another 1 case developing serious bilateral pleural effusion in the 3rd trimester, Other fetuses without SMs were born alive and with normal physical examinations at least 1 year after birth.

We also noted that the 5 cases with pathogenetic CNVs in our study were associated with SMs, including central nervous system (CNS), cardiovascular system and urinary system abnormalities as well as fetal hydrops and cystic hygroma. Bornstein et al recently reported the prevalence of pathogenic CNVs in 1980 high-risk and low-risk patients who had undergone prenatal diagnosis by CMA. As expected, pathogenic CNVs were much more common in cases of structural fetal abnormalities, with pathogenic CNVs rates of 5.9% for high-risk patients with fetal anomalies.^[4]^ Our study shows that the pathogenic CNVs rate in fetuses with a TNF associated with SMs is 16.1%, which is higher than that observed in Bornstein's study. This difference likely occurred because specific SMs that can cause a TNF have a higher risk of being associated with pathogenic CNVs. It is reasonable to use a CMA as a first-line test for fetuses with structural abnormalities, as the guidelines recommend.^[17,18]^

We observed de novo imbalanced chromosomal regions in fetus A14285. CMA revealed a 9.41 Mb deletion in chromosome 9p24.3-p23 and a 14.76 Mb duplication in 11p15.5-p15.2. Thus, parental karyotype analysis was performed; the cytogenetic result showed that the paternal karyotype was 46, XY, t(9,11)(p24,p15), and balanced chromosomal translocations can explain the CNV result. Additionally, we found that the CMA results of fetuses P6271 and P6425 did not match their phenotypes on ultrasound examination. The CMA of case P6271 showed a 17p12 deletion inherited from the non-phenotypic father, which is classified as pathogenic in the DECIPHER and ISCA databases. It overlaps with hereditary liability to pressure palsies (HNPP) syndrome in the DECIPHER database, which causes abnormalities in motor neurons and motor conduction block. This region includes 3 OMIM genes, among which the PMP22 gene (OMIM: 601097) results in morbidity. The PMP22 gene encodes a 22-kDa protein that comprises 2 to 5% of peripheral nervous system myelin and is expressed in the compact portion of essentially all myelinated fibers in the peripheral nervous system.^[19]^ Patients with CNVs matching this variant may have phenotypes such as intellectual disabilities, global developmental and motor delays, delayed speech and language development, generalized hypotonia, seizures, microcephaly, non-immune hydrops, abnormal facial shape, and others but not phenotypes such as short nasal bone, right hydronephrosis associated with ureteral dilatation, or gastric alveolar enlargement, as observed in fetus P6271. The CMA of case P6425 showed a 1.56 Mb deletion in 4q35.2 that overlapped with the deleted region in DECIPHER patient 286230. According to the DECIPHER database, patients with CNVs matching this variant experience symptoms such as intellectual disability, global developmental delays, delayed speech and language development, micrognathia, seizures, cleft palate, microcephaly, and muscular hypotonia. The 4q35.2 deletion contained 2 OMIM genes, including the FRG1 (FSHD region gene 1, OMIM:601278) and FGR2 genes (FSHD region gene 2, OMIM:609032). Both FRG1 and FGR2 are associated with facioscapulohumeral muscular dystrophy (FSHD; 158900), which is an autosomal dominant, inherited form of muscular dystrophy.^[20]^ FSHD is characterized by facial muscle weakness, shoulder weakness, hearing loss, abnormal heart rhythm, and loss of strength in abdominal muscles and typically has an onset in the first or second decade of life but can present later in life.^[21]^ These additionally identified pathogenic CNVs have different degrees of penetrance and expressivity, and based on the likelihood of a phenotype appearing and the existence of SMs, the parents decided to terminate the pregnancy. CMA is becoming the standard test for the prenatal evaluation of genomic imbalances, and there is likely to be an increase in the detection of unexpected diseases, especially for non-structural abnormalities, such as intellectual disability or late-onset disorders with unpredictable age of onset and severity. There is challenge for physicians and genetic counselors to address with patients, especially when a TNF is isolated without a specific structural abnormality. Thus, the development of consensus recommendations that are consistent with legal regulations and cultural beliefs are needed in the future to manage such incidental findings.

In our study, the rate of TNF cases associated with various SMs was high, up to 43%. As the NFT increased, the rate of SMs increased from 29.5% (NF measurement of 5–7.9 mm) to 76.4% (NF measurement ≥10 mm). The most commonly affected systems were the CNS and congenital heart disease (CHD) (Chart 1). Studies have shown that an increased NT measurement is correlated with a higher risk of chromosomal defects;^[22,23]^ however, the relationship between NF measurements and chromosomal defects have not been reported. Our study shows that an increase in NF measurement correlates with an increased risk of chromosomal defects, especially when the NF measurement is ≥10 mm; however, no statistically significant differences were found, likely due to the small sample size. In our study, 14 pregnant women chose termination because of various SMs, and 1 fetus died of premature delivery at 28 weeks of gestation despite normal CMA results. Case A11076 was found to have an isolated TNF in the second trimester with a normal CMA but developed serious bilateral pleural effusion, and the decision was made to terminate the pregnancy. If the diagnosis of a TNF is suspected after prenatal ultrasonography, careful sonographic evaluation of fetal anatomy to exclude additional malformations is recommended, and a series of ultrasounds may be necessary.

The Case series are susceptible to selection bias and our case series study reports on a series of patients with a TNF from a particular population (such as our clinic). Therefore, our study cases may not appropriately represent the wider population and we acknowledge this as limitation of the study.

## 5 Conclusion

A TNF is associated with a high risk of trisomy 21 and other clinically significant chromosomal abnormalities, including pathogenic CNVs, especially when the TNF is associated with structural abnormalities. CMA is recommended for prenatal diagnosis in TNF cases with one or more fetal structural abnormalities. The rate of structural abnormalities and adverse outcomes increases when the NF increases; especially when the thickness reaches 10 mm. Detailed ultrasound examination is recommended once a TNF is identified.

## Author contributions

Lushan Li, Fang Fu contributed equally to this work.

**Conceptualization:** Lushan Li, Fang Fu.

**Data curation:** Lushan Li.

**Formal analysis:** Lushan Li.

**Funding acquisition:** Can Liao.

**Investigation:** Lushan Li, Zequn liu.

**Methodology:** Ru Li.

**Project administration:** Fang Fu.

**Resources:** Lushan Li.

**Software:** Zequn liu.

**Supervision:** Fang Fu, Can Liao.

**Writing – original draft:** Lushan Li.

**Writing – review & editing:** Lushan Li.

Lushan Li orcid: 0000-0001-9450-6376.
